# USP15 Enhances Re-epithelialization Through Deubiquitinating EIF4A1 During Cutaneous Wound Repair

**DOI:** 10.3389/fcell.2020.00529

**Published:** 2020-06-26

**Authors:** Yixuan Zhao, Xin Huang, Zewei Zhang, Yifan Zhang, Guoyou Zhang, Tao Zan, Qingfeng Li

**Affiliations:** ^1^Department of Plastic and Reconstructive Surgery, Ninth People’s Hospital, Shanghai Jiao Tong University School of Medicine, Shanghai, China; ^2^Department of Plastic and Reconstructive Surgery, First Affiliated Hospital of Zhengzhou University, Zhengzhou, China

**Keywords:** epithelialization, USP15, deubiquitinating, post-translational modification, EIF4A1

## Abstract

Re-epithelialization is a fundamental process in wound healing that involves various cytokines and cells during cutaneous barrier reconstruction. Ubiquitin-specific peptidase 15 (*USP15*), an important member of the deubiquitinating enzymes (DUBs), removes ubiquitin chains from target proteins and maintains protein stability. However, the dynamic role of USP15 in epithelialization remains unclear. We aimed to investigate the regulatory function of USP15 in re-epithelialization. An excisional wound splinting model was established to evaluate the re-epithelialization rate in *Usp15* knockout (KO) mice. Coimmunoprecipitation (Co-IP) and mass spectrum analyses were performed to identify USP15-interacting proteins. RNA-sequencing was performed for transcriptome analysis in keratinocytes and uploaded into NODE database (http://www.biosino.org/node, accession numbers: OEP000770 and OEP000763). First, a significant delay in epithelialization was observed in the *Usp15* KO mice. Moreover, inhibition of cell migration and proliferation was observed in the USP15-silenced keratinocytes (HaCaTs). Moreover, we revealed for the first time that USP15 could interact with eukaryotic initiation factor 4A-1 (EIF4A1), thereby promoting translational efficacy in keratinocytes, which is essential for keratinocyte proliferation and migration. Conclusively, the USP15-EIF4A1 complex significantly accelerated re-epithelialization in wound healing. These observations helped elucidate the function and mechanisms of USP15 in modulating re-epithelialization in wound healing, providing a promising target for refractory wound treatment.

## Introduction

Wound healing is a complicated biological processes, involving the spatial and temporal synchronization of various cells and cytokines, with a distinct pattern in inflammatory, proliferative and remodeling phases (Tellechea et al., 2019). Deficiencies of homeostasis in the local microenvironment, including alterations in local physical forces, oxygen content, chemokines secretion and extracellular matrix remodeling, leads to impaired wound healing (Zhang et al., 2019). Delayed wound healing results in pain, infections, financial expenditures and loss of physical function, which affect over 40 million people (Miura et al., 2019). However, there has been no standard therapeutic approach to halt refractory wounds progression so far.

Keratinocytes in the basal layer of the skin play a major role in the maintenance of tissue homoeostasis and in skin repair in response to a cutaneous injury (Deshayes et al., 2018). During the re-epithelialization process, epithelial precursor cells migrate from the edges of the wound, proliferate and differentiate to create a new epidermis (Deshayes et al., 2018). Previous studies have demonstrated that keratinocyte function is essential in wound repair. For example, mice expressing human S100A2 exhibited delayed cutaneous wound repair, and the p53-S100A2 feedback loop impairs re-epithelialization in wound healing (Pan et al., 2018). Therapeutically, adipose stem cell-derived exosomes combined with hyaluronic acid significantly accelerated wound healing through promoting epithelialization and angiogenesis (Liu et al., 2019).

Ubiquitin-specific peptidase 15 (*USP15*), an important member of deubiquitinating enzymes (DUBs), removes ubiquitin chains from target proteins and promotes protein stability (Villeneuve et al., 2013). A previous study has shown that USP15 interacts with receptor-phosphorylated SMAD proteins (R-SMADs) and deubiquitinates transforming growth factor-β (TGF-β) receptor 1 (TBR1), thereby promoting protein stability (Inui et al., 2011). In addition, USP15 participates in diverse pathophysiological processes, such as regulating neuroinflammation, T cell activation and tumourigenesis (Eichhorn et al., 2012; Zou et al., 2014; Torre et al., 2017). Our previous study showed that USP15 plays a key role in the activation of the TGF-β signaling pathway in human dermal fibroblasts (HDFs) (Zhao et al., 2019). However, the role of USP15 in keratinocytes and re-epithelialization remains unclear.

Our research thus aimed to investigate the dynamic function of USP15 in re-epithelialization. USP15 was mainly distributed in keratinocytes, and a significant delay in epithelialization was observed in the *Usp15* knockout (KO) mice. In addition, inhibition of cell migration and proliferation was observed in the USP15-silenced keratinocytes. Moreover, for the first time, we revealed that USP15 could interact with and deubiquitinate eukaryotic initiation factor 4A-1 (EIF4A1), thereby promoting translational efficacy in keratinocytes. Taken together, these observations help elucidate the function of the USP15-mediated modulation of re-epithelialization during wound healing, providing a novel promising target for refractory wound treatment.

## Materials and Methods

### *Usp15* Knockout Mice

The animal experiments were approved by the Independent Committee of Shanghai Ninth People’s Hospital and conducted in accordance with the guidelines established by the National Health and Family Planning Commission of China. *Usp15* wt and *Usp15*−/− mice with a C57BL/6 background were maintained and bred under standard pathogen-free conditions and genotyped as previously described (Zhao et al., 2019). Eight- to ten-week-old male *Usp15* wild-type mice and *Usp15*−/− control littermates (offspring from heterozygote breeding) were used for the experiments. Only healthy mice without any inflammatory bowel disease were included in the study.

### Excisional Wound Splinting Model

Mice were anesthetized by an intraperitoneal injection of Ketanest/Rompun. The back was shaved, and two full-thickness cutaneous in 6mm diameter were generated using a standard biopsy punch. The 2 wounds on the back of each animal were at least 10 mm apart from each other. The wound was then sutured and fixed by a glued rubber ring. The mice were sacrificed on appropriate days, and an area of 8 mm in diameter, which included the complete epithelial margins, was excised. The wounds were bisected in the caudocranial direction, and the tissue was either fixed overnight in 4% paraformaldehyde or immediately frozen in liquid nitrogen. Histological analysis was performed on serial sections from the central portion of the wound.

### Immunofluorescence (IF) and Immunohistochemistry (IHC)

Wound beds surrounded by a margin of non-wounded skin were collected at days 0, 1, 3, 5, and 7 post wounding. The samples were fixed overnight in 4% paraformaldehyde at 4°C. The tissues were processed through graded ethanol solutions and embedded in paraffin blocks using standard protocols. The tissue sections (6 μm) were stained with haematoxylin and eosin. For the IHC and IF assays, the sections were incubated with primary antibody against USP15 (Abcam, ab4850, 1:200) and EIF4A1 (Abcam, ab31217, 1:200) diluted in blocking solution overnight at 4°C. After incubation with horseradish peroxidase-conjugated secondary antibody (IHC) or IF (anti-rabbit, 546 nm; anti-mouse, 488 nm), the sections were counterstained with haematoxylin and developed with diaminobenzidine.

### Western Blot Analysis

Cells were harvested at indicated times and rinsed twice with PBS. The cell extracts were prepared using lysis buffer and centrifuged at 13,000 *g* for 30 min at 4°C. The protein samples were separated by sodium dodecyl sulphate–polyacrylamide gel electrophoresis (SDS-PAGE) in 4–20% (wt/vol) polyacrylamide gels and transferred to polyvinylidene fluoride membranes. After the membranes were blocked with 5% BSA for 2 h at room temperature, they were incubated with 1.0 μg/mL antibody in 5% BSA overnight at 4°C. The membranes were then incubated with a secondary antibody conjugated to horseradish peroxidase. The signals were detected by electrochemiluminescence reagent. Protein bands were visualized in Amersham Imager 600 detection system (GE Chalfont, United Kingdom).

### RNA-Sequencing (RNA-seq)

Total RNA was extracted from the keratinocyte cell line (HaCaT) after silencing USP15 and EIF4A1 using TRIzol reagent (Invitrogen, Carlsbad, CA, United States). We confirmed the RNA integrity by using a 2100 Bioanalyzer (Agilent Technologies, United States). We measured the RNA concentration in a Qubit 2.0 fluorometer by using the Qubit RNA Assay Kit (Life Technologies, Carlsbad, CA, United States). We prepared the libraries from 100 ng of total RNA using an Illumina TruSeq RNA Sample Prep Kit (San Diego, CA, United States). The libraries were sequenced using the Illumina HiSeq 2500 platform (San Diego, CA, United States). The mRNA levels of the unigenes identified using TopHat v2.0.9 and Cufflinks were normalized by the Fragments Per Kilobase of exon model per Million mapped reads (FPKM), and the log2-fold changes between two samples were tested statistically to determine whether an individual gene’s expression was altered significantly. We used the criteria of false discovery rate (FDR) < 0.01 and fold changes <0.25 or >4.0 (<−2 or >2 in log2 ratio value, *p*-value < 0.05) to identify the differentially expressed genes. The value of gene expression was listed in Supplementary Table S2 (after silencing USP15) and Supplementary Table S3 (after silencing EIF4A1).

### Cell Counting Kit-8 (CCK-8) Assay

Cell proliferation was assessed by Cell Counting Kit8 (Dojindo, Tokyo, Japan) following the manufacturer’s protocals. Briefly, cells were seeded in triplicate in 96-well plates at a density of 2000 cells/100 μL. CCK-8 solution was added at the indicated time points to detect the absorbance at 450 nm.

### Colony Formation Assay

A volume of 2 mL of complete DMEM medium containing 2000 cells was placed in each well of a six-well plate. The plate was stained with 0.25% crystal violet after 2 weeks.

### Transwell Assay

A 24-well transwell system with polycarbonate filters (8-μm pores, Millipore, Burlington, MA, United States) was used. The upper compartment contained 10,0000 cells suspended in the appropriate medium with 2% FBS which was harvested 24 h later; the lower chamber contained 10% FBS. The upper cells in the chamber were removed, whereas those that migrated to the other side were stained with 0.25% crystal violet and photographed.

### Wound Healing Assays

Cells were seeded into 6-well dishes and grown to confluence. A sterilized 1-mL pipette tip was used to generate a scratch through the diameter, and the debris was washed away. A total of 8 areas were selected randomly in each well at 40x magnification, and the cells in three wells of each group were quantified in each experiment.

### Coimmunoprecipitation

For silver staining and mass spectrum (MS) analysis, coimmunoprecipitation was performed according to the Pierce^TM^ Co-Immunoprecipitation Kit (Thermo Fisher, United States, 26149). Briefly, 20 μg of anti-USP15 antibody (Proteintech, United States, 14354-1-AP) was coupled to the resin. Cellular lysate was then prepared using IP lysis buffer (25 mM Tris-HCl pH 8.0, 200 mM NaCl, 5 mM MgCl_2_ and 1 mM DTT) and incubated with coupled resin at 4°C overnight. The resins were then washed with IP lysis buffer and eluted with IP elution buffer (0.2 M glycine, pH 3.0). Other coimmunoprecipitation experiments were performed using the Pierce^TM^ Classic Magnetic IP/Co-IP kit (Thermo Fisher, United States, 88804). The lysate was first prepared by suspending the cells in IP lysis buffer for 15 min at 4°C. Immunoprecipitation was performed with 1 mg of protein and 10 μg of anti-EIF4A1 antibody in 500 μL of IP lysis buffer at 4°C overnight. The reaction mixtures were incubated with Protein A and Protein G Magnetic Beads (50 μL) at 4°C for 1 h on a rotator. The immunoprecipitated complexes were washed twice with IP wash buffer. The washed beads were incubated with 5× reduction loading buffer and boiled at 100°C for 5 min. The proteins released from components of the complexes were examined by SDS-polyacrylamide gel electrophoresis (PAGE) and western blotting with anti-USP15 antibodies.

### MS Analysis

Half of each peptide sample was separated and analyzed with a Nano-HPLC coupled to a Q-Exactive mass spectrometer (Thermo Finnigan). Separation was performed using a reversed-phase column (100 μm, ID × 15 cm, Reprosil-Pur 120 C18-AQ, 1.9 μm). The mobile phases were H_2_O with 0.1% FA and 2% ACN (phase A) and 80% ACN and 0.1% FA (phase B). Separation of the sample was executed with a 120-min gradient at a 300 nL/min flow rate. Gradient B was as follows: 8–35% for 92 min, 35–45% for 20 min, 45–100% for 2 min, 100% for 2 min, 100–2% for 2 min and 2% for 2 min. Data-dependent acquisition was performed in the profile and positive mode with the Orbitrap analyser at a resolution of 70,000 (200 m/z) and m/z range of 350–1400 for MS1; for MS2, the resolution was set to 17,500 (200 m/z). The top 10 most intense ions were fragmented by higher energy collisional dissociation (HCD) with a normalized collision energy (NCE) of 28% and isolation window of 2 m/z. The dynamic exclusion time window was 30 s.

### Cell Cycle Analysis

A total of 10^6^ cells were collected and fixed overnight in 70% ethanol at −20°C. The fixed cells were centrifuged and washed three times with 10 mL of PBS, resuspended in 500 μL of propidium iodide (BD-Pharmingen, United States, 550825) and incubated in the dark at room temperature for 15 min. The cells were analyzed by flow cytometry.

### Ubiquitination Assay

The lysate was first prepared by suspending the cells in IP lysis buffer for 30 min at 4°C. Immunoprecipitation was performed with 1 mg of protein and 10 μg of anti-EIF4A1 antibody in 500 μL of IP lysis buffer at 4°C overnight. The reaction mixtures were incubated with Protein A and Protein G Magnetic Beads (50 μL) at 4°C for 1 h on a rotator. The immunoprecipitated complexes were washed twice with IP wash buffer. The washed beads were incubated with 5× reduction loading buffer and boiled at 100°C for 5 min. The proteins released from components of the complexes were examined by SDS-polyacrylamide gel electrophoresis (PAGE) and western blotting with anti-Ub antibodies (Cell Signalling Technology, #3933).

### Statistical Analysis

The results are expressed as the mean ± SEM. Unpaired *t*-tests were conducted to identify significant differences (*p* < 0.05) in the wound-healing and scratch assay experiments. GraphPad Prism (GraphPad Software, San Diego, CA, United States) software was used for this analysis.

## Results

### Delayed Re-epithelialization in the *Usp15* Knockout Mice

**FIGURE 1:**
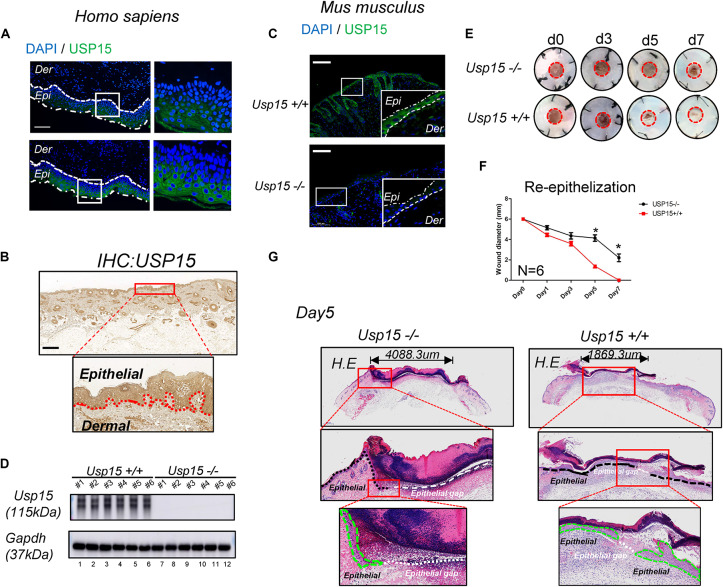
Delayed re-epithelialization in USP15–/– mice. **(A,B)** Immunofluorescence **(A)** and immunohistochemistry **(B)** of USP15 in human normal full-layer cutaneous tissue. Green: USP15; Blue: DNA; Scale bar: 50 μm. **(C)** Immunofluorescence of Usp15 in mouse normal cutaneous tissue. Green: Usp15; Scale bar: 50 μm. **(D)** Western blot assays were performed to measure Usp15 expression in the *Usp15*–/– animals and their wild-type littermates. Numbers of mice in each group: 6. **(E,F)** An excisional wound splinting model was established to evaluate the re-epithelialization rate in the USP15 knockout (KO) mice. Red circle: 6 mm diameter. *p < 0.05. Numbers of mice in each group: 6. **(G)** Haematoxylin-eosin staining revealed the epidermal gap in the excisional wound splinting model of the USP15 KO mice and their wild-type littermates. Black dash line: basal cell. Green dash area: regenerated epithelial. White dash line: the gap between regenerated epithelium of the wound area.

First, we tested USP15 expression in the full layer of human cutaneous tissue. Both IF (Figure 1A) and IHC (Figure 1B) showed that the USP15 protein signal was enriched in keratinocytes, while other cells presented weak USP15 expression. Furthermore, *Usp15* knockout mice were used in our study. Similarly, Usp15 was also distributed in the keratinocytes in full layer of mouse cutaneous tissue while presented weak signal in Usp15 KO mice (Figure 1C). We then confirmed that the USP15 protein expression in keratinocytes was indeed silenced in the *Usp15* knockout mice (Figure 1D, lanes 7–12). We then established an excisional wound splinting model and observed a significant delay in re-epithelialization in the *Usp15* knockout mice (Figures 1E,F). In the wound healing process, the gap (white dashed line) would be closed by the proliferation and migration of keratinocytes (green dashed line). Furthermore, we examined the epithelium gap of the wound model was significantly increased in *Usp15* knockout mice (Figure 1G).

### Loss of USP15 Attenuated the Proliferation and Migration of Keratinocytes

**FIGURE 2:**
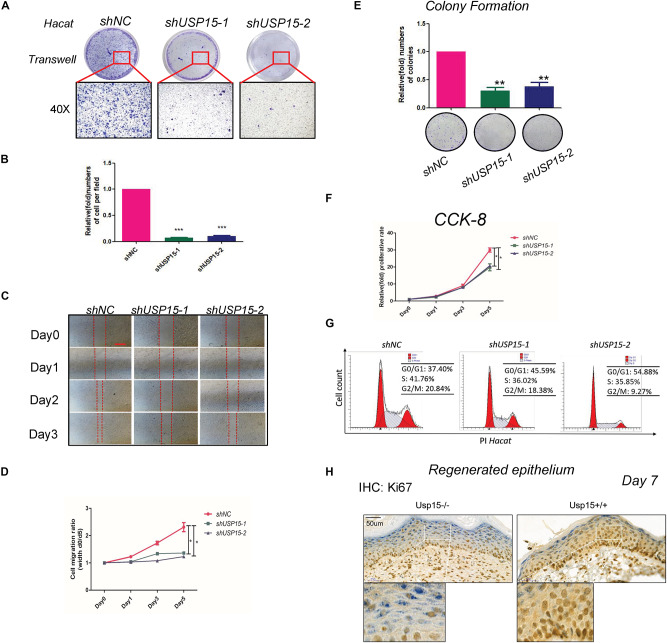
Loss of USP15 inhibited proliferation and migration *in vitro*. **(A)** A transwell assay was performed to determine the migratory ability of the *USP15*-silenced keratinocytes (HaCaT). **(B)** Quantification of the numbers of transferred cells. The colony number in the empty vector group was set as 100%. All experiments were performed in triplicate, and the relative cell numbers are shown as the mean ± SEM. *p < 0.05, **p < 0.01, ***p < 0.001. **(C)** A wound healing assay was performed to determine the migratory ability of the *USP15*-silenced keratinocytes (HaCaT). Scale bar: 50 μm. **(D)** Quantification of the wound recovery rate in the wound healing assay. All experiments were performed in triplicate, and the relative cell migration ratio is shown as the mean ± SEM. *p < 0.05. **(E)** A colony formation assay was performed to determine the colony formation ability of the USP15-silenced cells. The colony number in the shNC group was set as 100%. All experiments were performed in triplicate, and the relative colony formation rates are shown as the mean ± SEM. *p < 0.05, **p < 0.01. **(F)** A CCK-8 assay was performed to determine the proliferative rate of the USP15-silenced cells. The OD value of Day 0 was set as 100%. *p < 0.05. **(G)** Cell cycle analysis by flow cytometry was performed to determine the percentage of cells in the different cell cycle phases. The *X*-axis represents the FL2 channel-captured PI staining signals, and the *Y*-axis represents the cell counts. **(H)** Immunohistochemistry of Ki67 in the regenerated keratinocyte in *Usp15* knockout mice and their wild-type littermates at 7 days after establishing excisional wound splinting model. Scale bar: 50 μm.

We next explored the regulatory function of USP15 in keratinocytes. After transfecting lentiviruses with short hairpin sequences for USP15 (shUSP15) and the negative control (shNC), we observed the EGFP signal after transfection (Supplementary Figure S1A, panels 2–4). Moreover, we observed a significant reduction in the mRNA (Supplementary Figure S1B, upper panel) and protein expression of USP15 (Supplementary Figure S1B, lower panel). Transwell assays demonstrated that the upper layer cells transferred through an 8 μm hole decreased after inhibition of USP15 (Figures 2A,B). The wound healing assay showed a significant delay in the wound recovery rate in the USP15-silenced group (Figures 2C,D). Moreover, the cells also formed fewer and smaller colonies in the USP15 knockdown group than the control group (Figure 2E). The CCK-8 assay proved that the cellular proliferative rate was suppressed in the USP15-inhibited cells (Figure 2F). Flow cytometric assays showed a decreased percentage of cells in S phase after suppression of USP15 expression (Figure 2G). Notably, the difference of two shRNAs in flow cytometric assay could be due to different silencing efficiency of USP15. In addition, it could be also referred from unexpected ‘off-target’ silencing by shRNAs, which is reason why we performed two shRNAs in our study. Although it is different between two shRNAs groups, however, compare to control group, they all presented with decreased percentage of S phage and increased percentage of G0/G1 phase. More importantly, re-generated keratinocyte of *Usp15* KO cutaneous tissue presented decreased positive rate of Ki67 than wild-type group (Figure 2H). Taken together, these experiments showed that USP15 was vital in the proliferation and migration of keratinocytes, either *in vitro* or *in vivo*.

### Transcriptome Profiling in the USP15-Silenced Cells

**FIGURE 3:**
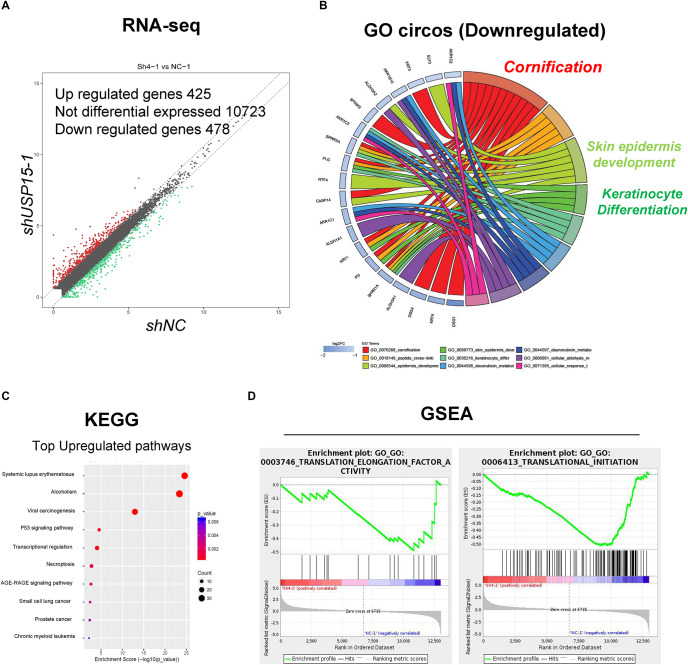
Transcriptional profiling in the USP15-silenced cells. **(A)** Volcano plots of differentially expressed genes. The red points denote the significantly upregulated genes, and the green points denote the significantly downregulated genes. Fold change > 1.5, *p* < 0.05. **(B)** A Gene ontology analysis and a Circos plot demonstrated that the differentially expressed genes were mainly associated with epidermal processes. The GO assay was performed in http://geneontology.org/. **(C)** A Kyoto Encyclopedia of Genes and Genomes assay illustrated pathways mainly involved in USP15 silencing. The KEGG assay was performed in https://www.kegg.jp/. **(D)** A Gene Set Enrichment analysis was performed to illustrate the translation-related pathway intensity after USP15 silencing. The GSEA assay was performed in https://www.gsea-msigdb.org/gsea/index.jsp.

To further explore the detailed mechanism of the regulatory role of USP15 in keratinocytes, we performed transcriptional screening after USP15 inhibition. An RNA-seq assay demonstrated that USP15 silencing led to the upregulation of 425 genes and downregulation of 475 genes in HaCaT cells (Figure 3A, fold change > 1.5, *p* < 0.05, accession number: OEP000770)^1^. Gene Ontology (GO) analyses and Circos plots showed that the main differentially expressed genes were associated with epidermal processes, such as cornification, skin epidermal development and keratinocyte differentiation (Figure 3B), while upregulated genes were mainly associated with transcriptional regulation (Supplementary Figure S2A), such as nucleosome assembly, H3K27 tri-methylation and RNA polymerase II guided transcription. The Kyoto Encyclopedia of Genes and Genomes (KEGG) analysis demonstrated that the top upregulated pathways were associated with immunity and regulation of carcinogenesis (Figure 3C), while downregulated pathways were associated with infection and metabolic regulation (Supplementary Figure S2B). Notably, our group previously demonstrated that USP15 could activate TGF-β signaling pathway activity in dermal fibroblasts. However, in keratinocytes, the TGF-β signaling pathway was not downregulated after USP15 was silenced (Supplementary Figure S3A). Moreover, through a Gene Set Enrichment Analysis (GSEA), we found that the translation-related process was significantly downregulated after suppressing USP15 (Figure 3D).

### USP15 Interacted With EIF4A1

**FIGURE 4:**
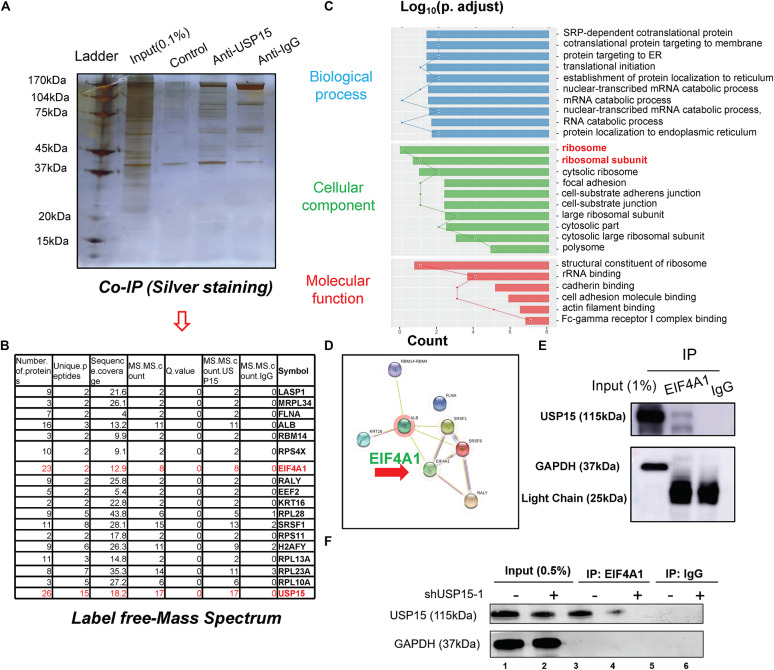
The interactome analysis of USP15. **(A)** A coimmunoprecipitation assay was performed to identify the USP15-interacting proteins. The eluted protein was identified by silver staining. **(B)** A mass spectrum analysis was performed, and 18 specified USP15-interacting proteins were identified. **(C)** A Gene Ontology assay demonstrated that the differentially expressed genes were mainly distributed in ribosomes. The GO assay was performed in http://geneontology.org/. **(D)** A protein interaction network assay was performed to identify the central proteins in the USP15 interactome. This assay was performed in https://string-db.org/. **(E)** An immunoprecipitation assay was performed to illustrate the interaction between EIF4A1 and USP15. EIF4A1 was used as a bait. The GAPDH protein presented a strong band in input group while IgG light chain only presented in IP group. **(F)** An immunoprecipitation assay was performed after silencing USP15. EIF4A1 was used as a bait. The GAPDH protein presented a strong band in input group while absent in IP group.

To underline the detailed mechanism of USP15 in translational regulation, we performed coimmunoprecipitation, and the lysate was identified by silver staining (Figure 4A). A mass spectrum analysis identified 18 proteins that could specifically bind USP15 (Figure 4B and Supplementary Figure S4A). These 18 proteins were identified in three replicates of mass spectrum using anti-USP15, however, no signal was detected in anti-IgG group (Supplementary Figure S4B). GO assays showed that these specific binding proteins were mainly distributed in the ribosome (Figure 4C and Supplementary Figure S5A). Furthermore, through a protein interaction network, we found that EIF4A1 may play a central role in USP15-guided translational regulation (Figure 4D). In addition, a western blot assay proved that the USP15 protein could be pulled down in the anti-EIF4A1 group, which indicated a direct interaction between EIF4A1 and USP15 (Figure 4E). It should be explained that EIF4A1 protein was 48 kDa, which is similar to IgG heavy chain (50 kDa). We were unable to distinguished these two bands after IP assay if we used USP15 as a bait. Moreover, after silencing USP15, the global protein USP15 has been largely reduced (Figure 4F, lane 1) and weaker interaction signal was then identified (Figure 4F, lane 4).

### USP15 Deubiquitinated EIF4A1 and Enhanced Its Stability *in vitro* and *in vivo*

**FIGURE 5:**
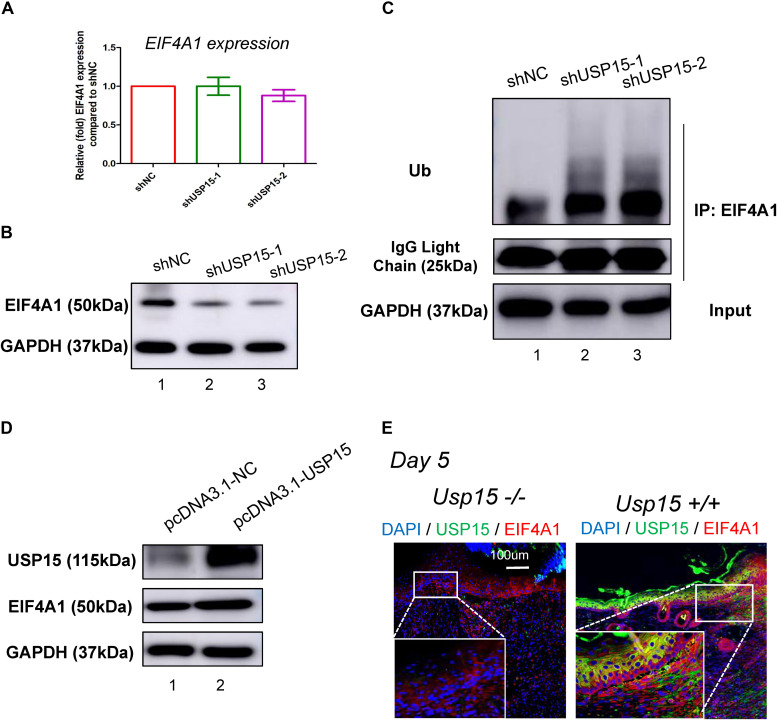
USP15 removed ubiquitin from EIF4A1. **(A)** Real-time PCR was performed to measure the EIF4A1 mRNA expression in the USP15-silenced keratinocytes (HaCaT). **(B)** A western blot assay was performed to identify the EIF4A1 protein expression in the USP15-silenced keratinocytes (HaCaT) cells. **(C)** A coimmunoprecipitation assay was performed to measure the ubiquitination level of EIF4A1 after inhibiting USP15. **(D)** A western blot assay was performed to identify the EIF4A1 protein expression in the USP15-overexpressed keratinocytes (HaCaT) cells. pcDNA3.1 was used as vector for overexpression. pcDNA3.1-NC was referred as control. **(E)** Immunofluorescence of USP15 and EIF4A1 in the *Usp15* knockout mice and their wild-type littermates. Scale bar: 200 μm.

Since USP15 could interact with EIF4A1, we then investigated whether EIF4A1 expression could be regulated by USP15. We found that the mRNA level of EIF4A1 remained unchanged after silencing USP15 (Figure 5A). However, a significant reduction in the EIF4A1 protein level was observed after knocking down USP15 (Figure 5B, lanes 2–3). These results indicated that USP15 could enhance EIF4A1 expression at the post-transcriptional level. Since USP15 is a DUB, we tested the ubiquitination level after silencing USP15. As expected, the ubiquitination level of EIF4A1 was significantly upregulated after inhibiting USP15 (Figure 5C). We then overexpressed Usp15 in HaCaT cells. We observed a significant increase of Usp15 expression in both mRNA (Supplementary Figure S6A) and protein (Figure 5D, 1st panel) level. Moreover, we have observed EIF4A1 protein expression was significantly upregulated (Figure 5D, 2nd panel) while the RNA expression of EIF4A1 remain unchanged (Supplementary Figure S6B) which demonstrated that USP15 could interact with and deubiquitinate EIF4A1, thus promoting EIF4A1 protein stability. Furthermore, a weak fluorescent signal of EIF4A1 was observed in the USP15−/− mice, indicating that USP15 could also promote EIF4A1 protein stability *in vivo* (Figure 5E).

### EIF4A1 Promoted the Proliferation, Migration and Translation of Keratinocytes

**FIGURE 6:**
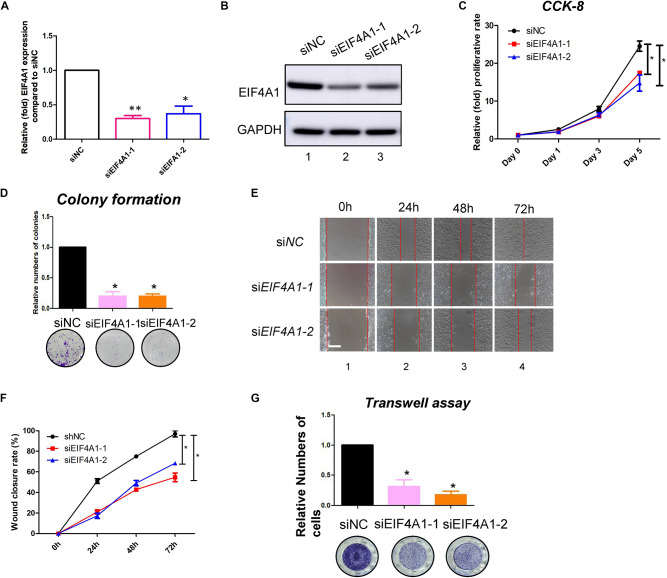
EIF4A1 promoted the cell growth and migration in keratinocytes. **(A)** Real-time PCR was performed to measure the EIF4A1 mRNA expression in the EIF4A1-silenced HaCaT cells. Scramble-siRNAs served as a negative control (siNC). *p < 0.05, **p < 0.01. **(B)** A western blot assay was performed to identify the EIF4A1 protein expression in the EIF4A1-silenced HaCaT cells. GAPDH was referred as endogenous control. **(C)** A CCK-8 assay was performed to determine the proliferative rate of the EIF4A1-silenced cells. The OD value of Day 0 was set as 100%. *p < 0.05. **(D)** A colony formation assay was performed to determine the colony formation ability of the EIF4A1-silenced cells. The colony number in the shNC group was set as 100%. All experiments were performed in triplicate, and the relative colony formation rates are shown as the mean ± SEM. *p < 0.05. **(E)** A wound healing assay was performed to determine the migratory ability of the EIF4A1-silenced keratinocytes (HaCaT). Scale bar: 50 μm. **(F)** Quantification of the wound recovery rate in the wound healing assay. All experiments were performed in triplicate, and the relative cell migration ratio is shown as the mean ± SEM. *p < 0.05. **(G)** A transwell assay was performed to determine the migratory ability of the EIF4A1-silenced cells. Quantification of the numbers of transferred cells is shown in the upper panel. The colony number in the empty vector group was set as 100%. All experiments were performed in triplicate, and the relative cell numbers are shown as the mean ± SEM. *p < 0.05.

**FIGURE 7:**
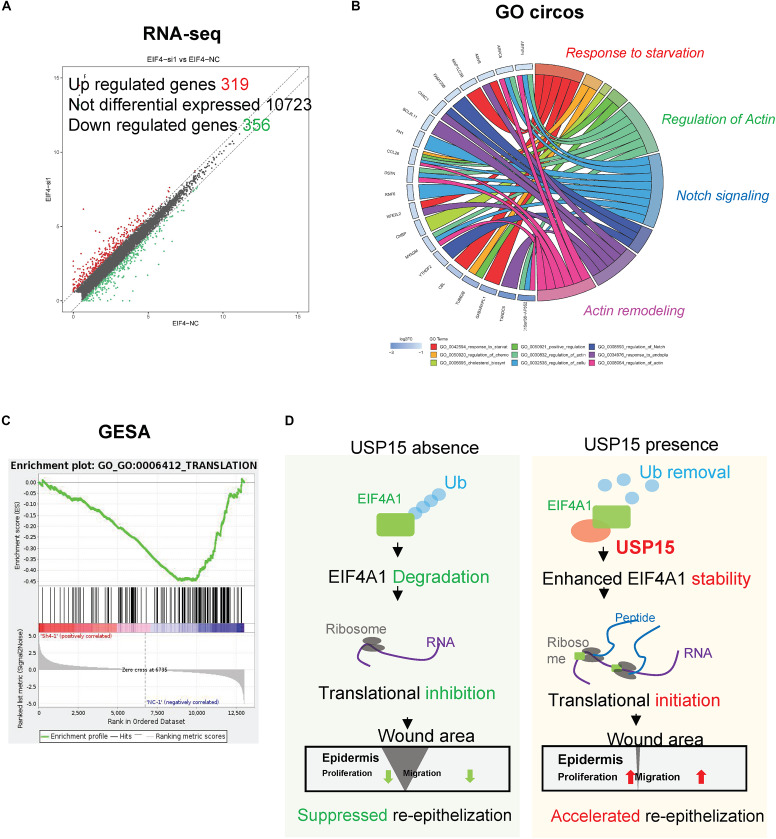
Transcription profiling in the EIF4A1-silenced cells. **(A)** Volcano plots of differentially expressed genes. The red points denote 319 significantly upregulated genes, and the green points denote 356 downregulated genes. Fold change > 1.5, *p* < 0.05. **(B)** A Gene Ontology assay and Circos plot demonstrated that the differentially expressed genes were mainly associated with cytoskeleton remodeling. The GO assay was performed in http://geneontology.org/. **(C)** A Gene Set Enrichment analysis illustrated that the translation signaling pathway was significantly inhibited after EIF4A1 silencing. This assay was performed in https://string-db.org/. **(D)** Schematic of the study. Silencing *USP15/EIF4A1* attenuates the translation initiation process and results in dysfunctional re-epithelialization. USP15 directly deubiquitinates EIF4A1 to promote wound healing in keratinocytes.

EIF4A1 is a key factor in translational initiation; however, the role of EIF4A1 in keratinocytes remains unclear. Thus, we inhibited EIF4A1 expression with two siRNAs in HaCaT cells. Both siRNAs resulted in an ∼70% mRNA knockdown efficacy (Figure 6A). In addition, the EIF4A1 protein level was significantly downregulated after silencing EIF4A1 (Figure 6B). Furthermore, the EIF4A1-silenced cells presented a slower proliferative rate and formed smaller colonies than the control cells (Figures 6C,D). Moreover, both wound healing assays (Figures 6E,F) and Transwell assays (Figure 6G) demonstrated that cellular migration was significantly attenuated after knocking down EIF4A1. Most importantly, a transcriptome analysis was performed and demonstrated that there were 319 upregulated and 356 downregulated genes after silencing EIF4A1 (Figure 7A, see footnote 1, accession number: OEP000763). These altered genes were mainly involved in cellular starvation, cytoskeleton/actin remodeling and the notch signaling pathway (Figure 7B). Moreover, a significant reduction in the translation process was observed after interfering with EIF4A1, which indicates the essential role of EIF4A1 in translation (Figure 7C).

## Discussion

Re-epithelialization plays a vital role in wound healing, which involves numerous cytokines and cells during cutaneous barrier reconstruction (Mazzalupo et al., 2002; Jackow et al., 2016). USP15, an important DUB, removes ubiquitin chains from target proteins and promotes protein stability (Padmanabhan et al., 2018). Here, we demonstrated for the first time that a keratinocyte-expressed protein, USP15, deubiquitinated EIF4A1 and prevented its degradation, thereby enhancing the translation process and accelerating re-epithelialization both *in vitro* and *in vivo* (Figure 7D). These observations shed light on the novel regulatory role of USP15 in the re-epithelialization of wound healing, providing a promising target for refractory wound treatment.

Notably, USP15, a vital DUB that can remove ubiquitin from proteins, has been shown to be involved in DNA repair, TGF-β signaling pathways and mitophagy (Cornelissen et al., 2014). For instance, USP15 was reported to regulate transcription and DNA repair via deubiquitinating the histone H2B (Long et al., 2014). Our group previously demonstrated that USP15 could directly accelerate wound healing by stabilizing TBR1 and maintaining the TGF-β signaling pathway in fibroblasts (Zhao et al., 2019). However, the role of USP15 in keratinocytes was different from that in fibroblasts. In our study, we found that the TGF-β signaling pathway remained unchanged in keratinocytes, which indicated a novel function of USP15.

To explore the target protein of USP15, we performed a multi-omics analysis. RNA-seq demonstrated that USP15 was highly associated with translational regulation. Coimmunoprecipitation and MS analyses revealed that the interactome of the USP15 protein was mainly distributed in the ribosome, and EIF4A1 was a key factor in translational initiation. Here, we revealed for the first time that USP15 could enhance the translation process, indicating a novel function of the USP15/EIF4A1 complex.

Translation initiation is a rate-limiting and highly regulated process that requires diversified coordinated action of eukaryotic initiation factors (EIFs) (Wolf et al., 2010). The DEAD-box helicase eIF4A1 has been proven to unwind structured RNA elements within the 5′ untranslated region (5′UTR) to facilitate ribosome binding (Modelska et al., 2015; Peters et al., 2018). Alterations in EIF4A1 activity-modulating proteins expression have been observed in the tumourigenesis of melanoma (Joyce et al., 2017), breast cancer (Stoneley and Willis, 2015), and pancreatic cancer (Ma et al., 2019). However, the role of EIF4A1 in re-epithelialization remains unclear. Here, for the first time, we demonstrated that EIF4A1 was essential in the translational regulation, proliferation and migration of keratinocytes, which indicated a novel molecular function in wound healing and a promising target of refractory wounds.

Mostly, translational initiation factor family were ‘house-keeping genes’ and initiation of translation is the rate-limiting step in protein synthesis in all living cells (Mejias-Navarro et al., 2020). These factors have been proven to play a vital role in wound healing, tumorigenesis, cell stemness and epithelial-mesenchymal transition, etc. For example, elF2a is involved in DNA damage repair and could regulate autophagy in tumors (Arasi et al., 2019). elF3 family modulate of the hypoxia inducible factors (HIFs) and suppress tumorigenesis. elF4e has been identified to be involved in cutaneous wound healing (Schwarz et al., 2002). Moreover, EIF4A1 was identified to be significantly upregulated in a proteomic investigation of human burn wounds by 2D-difference gel electrophoresis (Wolf et al., 2010; Joyce et al., 2017). Conclusively, the initiation factors are ‘house-keeping genes,’ which are vital in protein homeostasis, it is hard to choose another initiation factor as control. We would like to further validate the role of other initiation factors in wound healing, which would warrant an individual study.

Skin wound healing requires diverse coordinated interactions across various cells, such as macrophages, activated T/B lymphocytes and fibroblasts (Schmidt and Horsley, 2013; Rognoni and Watt, 2018). To date, our group has only uncovered the functions of USP15 in fibroblasts and keratinocytes. Further investigations could explore the regulatory roles of USP15 in other cells. In addition, mice and humans show a fundamental difference in cutaneous wound healing (Dunn et al., 2013). Further studies should be performed to explore the possibility of treating chronic refractory wounds through supplementation with recombinant *USP15* protein or USP15-carrying adenovirus.

Thus, we concluded that silencing *USP15/EIF4A1* attenuates the translational initiation process and then results in dysfunctional re-epithelialization. Mechanistically, our research proves that USP15 directly deubiquitinates EIF4A1 to promote wound healing in keratinocytes. Because USP15 harbors druggable enzymatic activity as a member of the DUBs, it is considered a potential therapeutic target with vital clinical applications (He et al., 2017). Recombinant DUBs or DUB-based virus-related therapy could further be applied to accelerate re-epithelialization, whereas small molecule inhibitors that target DUBs may become a promising intervention for cutaneous overhealing-associated diseases, such as hypertrophic scars and keloids (Lee et al., 2010; Piao et al., 2015). Further experiments on the effects of overexpressing *USP15* (such as recombinant protein or virus-related therapy) will be performed in the near future to assess the function of USP15 in the treatment of refractory wounds.

## Data Availability Statement

Publicly available datasets were analyzed in this study. This data can be found here: RNA-seq data has been uploaded to National Omics Data Encyclopedia (NODE) database (http://www.biosino.org/node/project, accession number: OEP000770 for USP15 silencing and OEP000763 for EIF4A1 silencing).

## Ethics Statement

The animal study was reviewed and approved by Ethics Committee of Shanghai Ninth People’s Hospital.

## Author Contributions

YXZ and XH designed and performed the experiments and drafted the manuscript. YXZ and ZZ were responsible for the sample collection and data analysis. YFZ revised the manuscript. GZ provided the mice. QL, TZ, and GZ discussed and approved the manuscript. All the authors approved this manuscript.

## Conflict of Interest

The authors declare that the research was conducted in the absence of any commercial or financial relationships that could be construed as a potential conflict of interest.

## Footnotes

1 http://www.biosino.org/node

## Abbreviations

DUBs: deubiquitinating enzymes
ECM: extracellular matrix
EIFA1: eukaryotic initiation factor 4A-1
HDFs: human dermal fibroblasts
PTM: post-translational modification
R-SMADs: receptor-regulated SMADs
TBR1: TGF- β type I receptor
TGF- β: transforming growth factor- β
USP15: ubiquitin-specific peptidase 15
USP4: ubiquitin-specific protease 4.
