# Supplementary material for: USP15 Enhances Re-epithelialization Through Deubiquitinating EIF4A1 During Cutaneous Wound Repair
Source: Front Cell Dev Biol. 2020 Jun 26;8:529. doi: 10.3389/fcell.2020.00529 (PMC7332549; doi:10.3389/fcell.2020.00529)
Supplement: Supplementary file 1 [file Data_Sheet_1.doc]

**Supplementary files**

**USP15 enhances re-epithelization through deubiquitinating EIF4A1 during cutaneous wound repair**

Yixuan Zhao^1^*, M.D., Ph.D., Xin Huang^1^*, M.D., Zewei Zhang^1,2^, M.D., Guoyou Zhang^1^, M.D, Ph.D., Yifan Zhang^1^, M.D, Ph.D., Tao Zan^1#^, M.D., Ph.D., Qingfeng Li^1#^, M.D., Ph.D.

^1^Department of Plastic and Reconstructive Surgery, Ninth People’s Hospital, Shanghai Jiao Tong University School of Medicine, Shanghai, P.R. China

^2^Department of Plastic and Reconstructive Surgery, First Affiliated Hospital of Zhengzhou University, Zhengzhou, Henan Province, P.R. China

*These authors contributed equally to this report.

^#^These authors are co-corresponding authors of this report.

Corresponding authors:

Qingfeng Li, Department of Plastic and Reconstructive Surgery, Shanghai Ninth People’s Hospital, Shanghai Jiao Tong University School of Medicine, 639 Zhizaoju Road, Shanghai 200011, P.R. China.

E-mail: dr.liqingfeng@yahoo.com (Qingfeng Li); zantaodoctor@yahoo.com (Tao Zan).

**Running Title:** USP15 promotes re-epithelialization

**Figure legend**

Supplemental information includes 3 figures and 3 table.

**Supplementary Tables**

**Supplementary Table 1:** Primers, siRNAs, sgRNAs, shRNAs and antibodies used in the experiment.

**Supplementary Table 2:** Gene expression after silencing USP15.

**Supplementary Table 3:** Gene expression after silencing EIF4A1.

**Supplementary Figure 1: Establishing stable USP15 silenced cells.**

(**A**) GFP signal detection after transfecting lentivirus with an EGFP tag.

(**B**) mRNA (upper panel) and protein (lower panel) USP15 expression after lentivirus transfection.

**Supplementary Figure 2: Bioinformatics assay after silencing USP15.**

**(A):** A Gene ontology analysis and a Circos plot demonstrated that the upregulated expressed genes were mainly associated with transcriptional regulation after silencing USP15. The GO assay was performed in http://geneontology.org/.

(B): A Kyoto Encyclopaedia of Genes and Genomes (KEGG) assay illustrated upregulated pathways involved in USP15 silencing. The KEGG assay was performed in https://www.kegg.jp/.

**Supplementary Figure 3: TGF-β signalling pathway activity remain unchanged in keratinocytes.**

A Gene Set Enrichment analysis was performed to measure TGF-β signalling pathway activity in USP15 silenced cells.

**Supplementary Figure 4: USP15 specific binding proteins.**

(**A**) Three replicates of mass spectrum (MS) identified 18 common USP15 specifically binding proteins. (B) The signal of 18 proteins detected in MS.

**Supplementary Figure 5: GO analysis of** **USP15 specific binding proteins.**

A GO assay demonstrated that these USP15 binding proteins were mainly distributed in spliceosome and ribosomes.

**Supplementary Figure 6： Overexpression of USP15 using pcDNA3.1 vector.**

**(A-B)** A real-time PCR was performed to detect USP15 expression and EIF4A1 expression after overexpression USP15. **, p<0.01.

**Supplementary Table 1**

| **Primers** | |
| --- | --- |
| *USP15* | F: 5'-AAAACCTCGCTCCGGAAAGG-3' |
|  | R: 5'-CCACCTTTCGTGCTATTGG-3' |
| *GAPDH* | F: 5'-GGGAAGGTGAAGGTCGGAGT-3' |
|  | R: 5'-GGGGTCATTGATGGCAACA-3' |
| *EIF4A1* | F: 5'- TGTCTGCGAGCCAGGATTCCC -3' |
|  | R: 5'- AGATGCCACGGAGAAGGGACTC -3' |
| **siRNAs** | |
| siEIF4A1 | siRNA-1: 5’-GCCGUGUGUUUGAUAUGCUUATT-3’ |
|  | siRNA-2: 5’-CCUUGUAUCAAGGGUUAUGAUTT-3’ |
| ***Usp15* knockout mice** | |
| Usp15-S1 | sgRNA: 5'-CCTGGACCCATCGATAACTC-3' PAM: TGG |
| Usp15-S2 | sgRNA: 5'-AGCTGGGACAAATACCAGAT-3' PAM: GGG |
| **shRNAs** | |
| shUSP15-1 | shRNA-1: 5'-GATACAGAGCACGTGATTATT-3' |
| shUSP15-2 | shRNA-2: 5'-GGAATGGCCCAAATGGCATAC-3' |
| **Antibodies** | |
| EIF4A1/Eif4a1 | ab31217, Abcam; |
| USP15/Usp15 | 14354-1-AP, Proteintech; ab4850, Abcam |
| Ub | ab19247, Abcam, |
| GAPDH | ab181602, Abcam |
