# Supplementary material for: USP15 Enhances Re-epithelialization Through Deubiquitinating EIF4A1 During Cutaneous Wound Repair
Source: Front Cell Dev Biol. 2020 Jun 26;8:529. doi: 10.3389/fcell.2020.00529 (PMC7332549; doi:10.3389/fcell.2020.00529)
Supplement: Supplementary file 2 [file Image_1.pdf]

Fig.S1

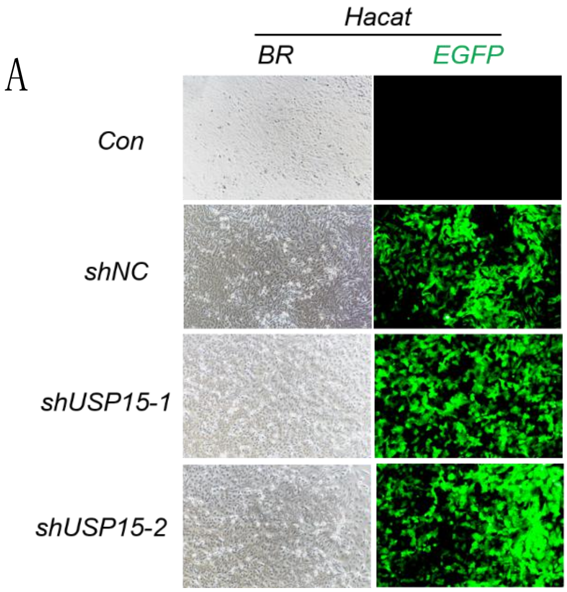
A
Hacat
BR
EGFP
Con
shNC
shUSP15-1
shUSP15-2

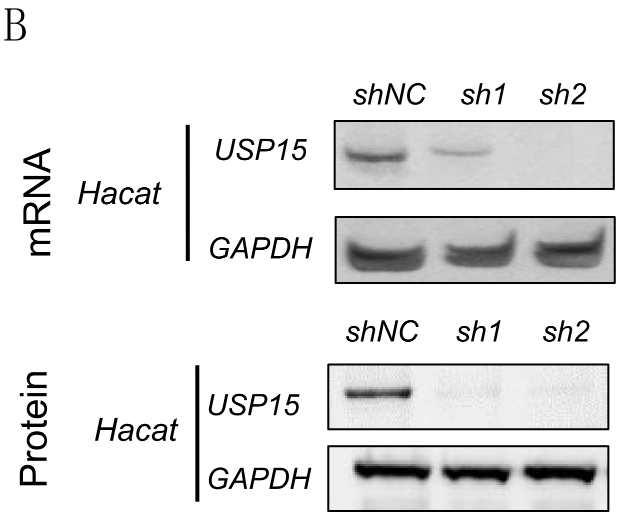
B
shNC
sh1
sh2
mRNA
Hacat
USP15
GAPDH
shNC
sh1
sh2
Protein
Hacat
USP15
GAPDH

Fig. S2

A

GO circus (Upregulated genes)

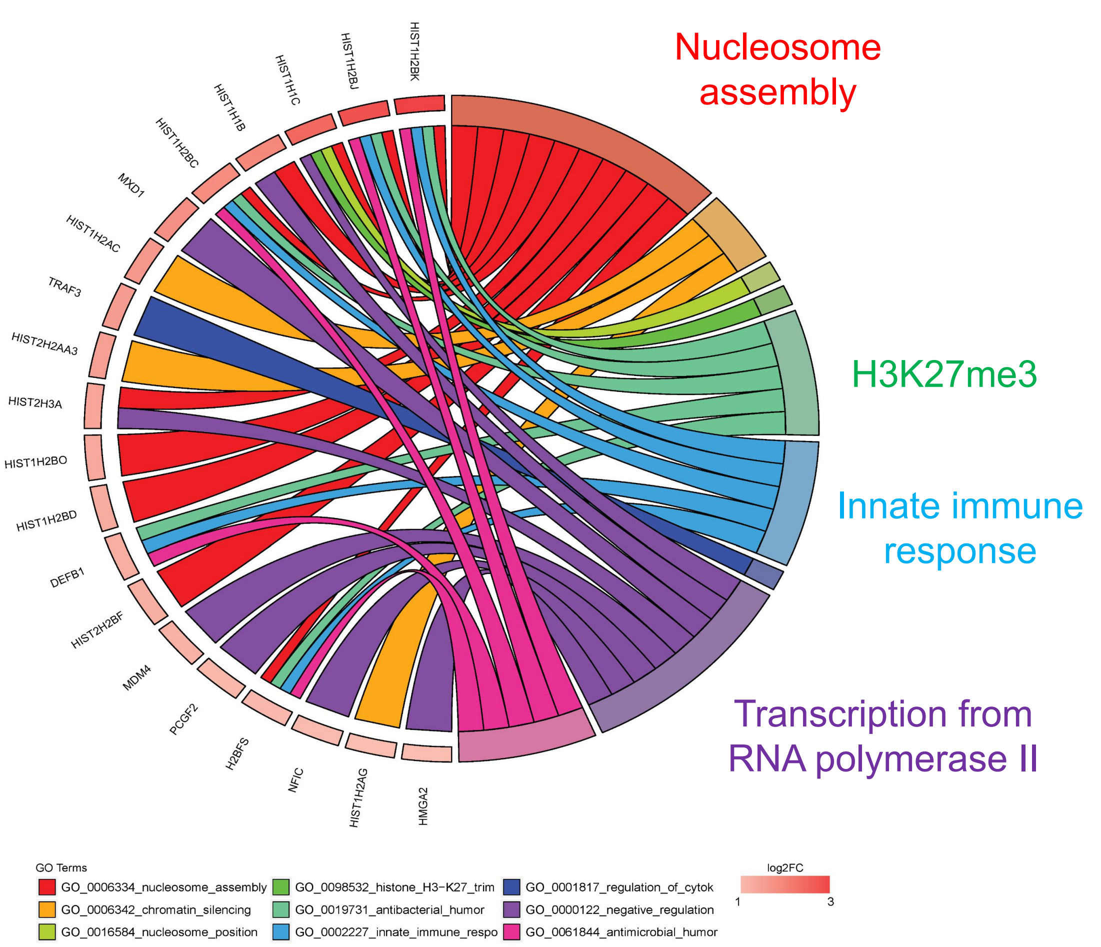

B

KEGG (Top downregulated genes)

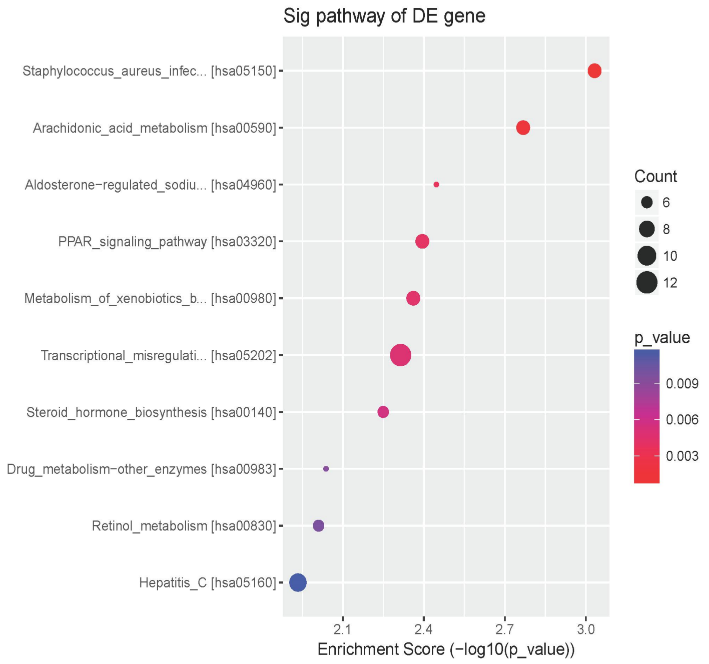

Fig. S3

GSEA

shUSP15 VS shNC

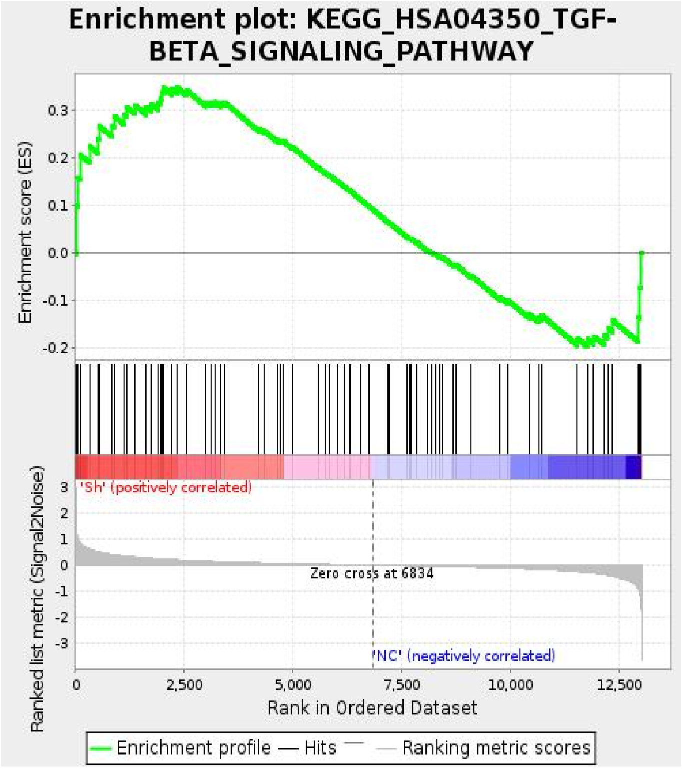

Fig. S4

A

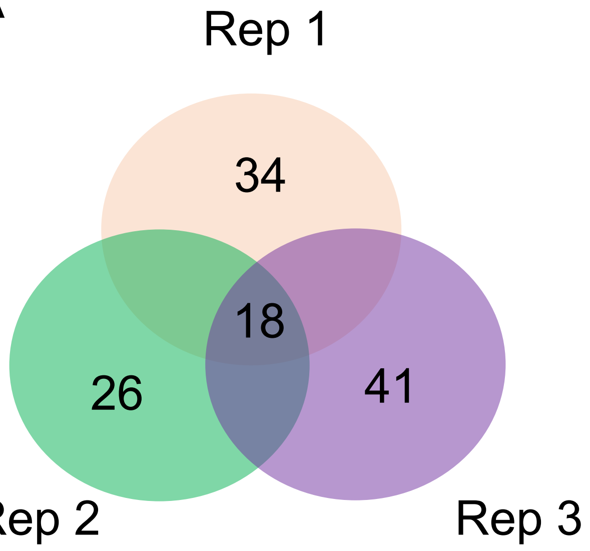

B

Rep 1		Rep 2		Rep 3		
MS.MS.count.USP15	MS.MS.count.IgG	MS.MS.count.USP15	MS.MS.count.IgG	MS.MS.count.USP15	MS.MS.count.IgG	**Symbol**
2	0	5	1	1	0	**LASP1**
2	0	2	0	1	0	**MRPL34**
2	0	3	0	2	0	**FLNA**
11	0	7	0	21	0	**ALB**
2	0	2	0	2	0	**RBM14**
2	0	5	0	3	0	**RPS4X**
8	0	6	0	4	0	**EIF4A1**
2	0	8	0	4	0	**RALY**
2	0	3	0	4	1	**EEF2**
2	0	2	0	2	0	**KRT16**
5	1	4	1	5	1	**RPL28**
13	2	23	2	13	2	**SRSF1**
2	0	11	0	2	0	**RPS11**
9	2	4	0	9	1	**H2AFY**
2	0	2	0	2	0	**RPL13A**
11	3	7	3	11	5	**RPL23A**
6	0	5	0	4	0	**RPL10A**
17	0	20	0	14	0	**USP15**

Label free-Mass Spectrum

Fig. S5

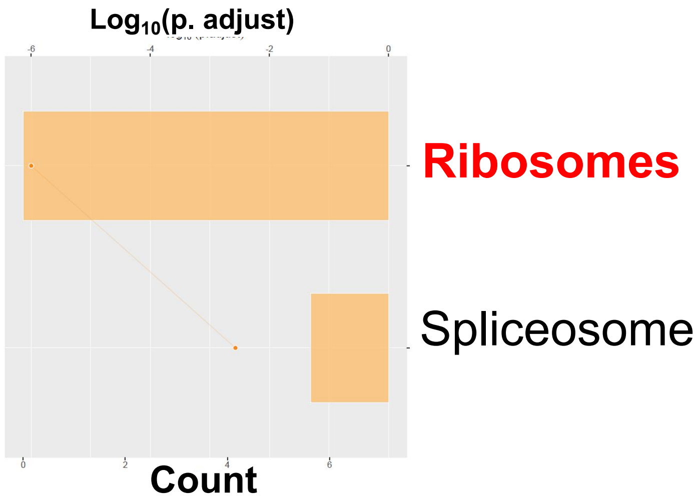

Fig. S6

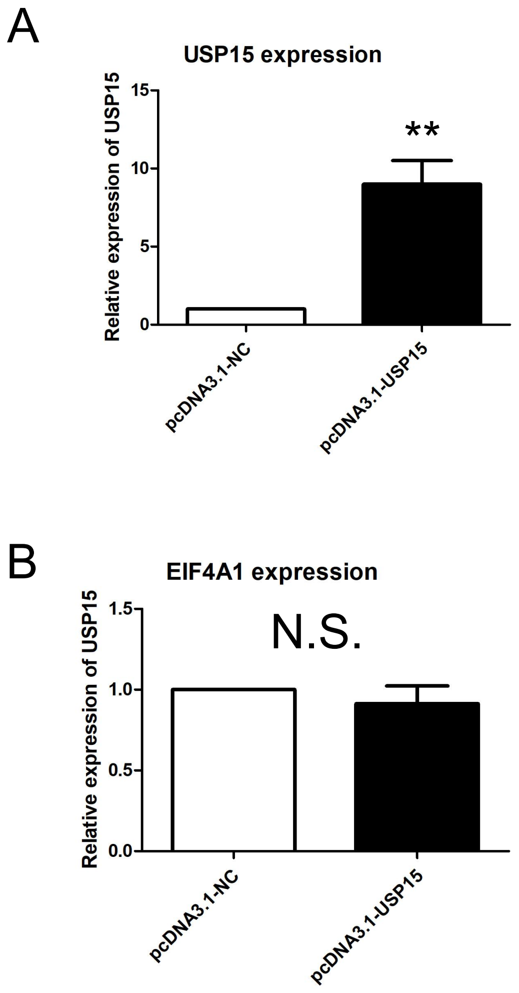
A
USP15 expression
Relative expression of USP15
15
10
5
0
**
pcDNA3.1-NC
pcDNA3.1-USP15
B
EIF4A1 expression
Relative expression of USP15
1.5
1.0
0.5
0.0
N.S.
pcDNA3.1-NC
pcDNA3.1-USP15
