# Supplementary material for: USP15 Enhances Re-epithelialization Through Deubiquitinating EIF4A1 During Cutaneous Wound Repair
Source: Front Cell Dev Biol. 2020 Jun 26;8:529. doi: 10.3389/fcell.2020.00529 (PMC7332549; doi:10.3389/fcell.2020.00529)
Supplement: Supplementary file 3 [file Table_1.docx]

**Supplementary Table 1**

| **Primers** | |
| --- | --- |
| *USP15* | F: 5'-AAAACCTCGCTCCGGAAAGG-3' |
|  | R: 5'-CCACCTTTCGTGCTATTGG-3' |
| *GAPDH* | F: 5'-GGGAAGGTGAAGGTCGGAGT-3' |
|  | R: 5'-GGGGTCATTGATGGCAACA-3' |
| *EIF4A1* | F: 5'- TGTCTGCGAGCCAGGATTCCC -3' |
|  | R: 5'- AGATGCCACGGAGAAGGGACTC -3' |
| **siRNAs** | |
| siEIF4A1 | siRNA-1: 5’-GCCGUGUGUUUGAUAUGCUUATT-3’ |
|  | siRNA-2: 5’-CCUUGUAUCAAGGGUUAUGAUTT-3’ |
| ***Usp15* knockout mice** | |
| Usp15-S1 | sgRNA: 5'-CCTGGACCCATCGATAACTC-3' PAM: TGG |
| Usp15-S2 | sgRNA: 5'-AGCTGGGACAAATACCAGAT-3' PAM: GGG |
| **shRNAs** | |
| shUSP15-1 | shRNA-1: 5'-GATACAGAGCACGTGATTATT-3' |
| shUSP15-2 | shRNA-2: 5'-GGAATGGCCCAAATGGCATAC-3' |
| **Antibodies** | |
| EIF4A1/Eif4a1 | ab31217, Abcam; |
| USP15/Usp15 | 14354-1-AP, Proteintech; ab4850, Abcam |
| Ub | ab19247, Abcam, |
| GAPDH | ab181602, Abcam |
